# Seasonal Effect on the Chemical Composition of *Lippia alba* Volatile Chemotypes

**DOI:** 10.1002/cbdv.71664

**Published:** 2026-08-31

**Authors:** Marcelino Santiago Barroso Neto, Erilene Nayrã Mendonça Brito Lopes, Marcia Ortiz Mayo Marques, Alex Aparecido Rosini Silva, Patricia de Oliveira Carvalho, Alexandra Christine Helena Frankland Sawaya

**Affiliations:** ^1^ Institute of Biology State University of Campinas Campinas Brazil; ^2^ Agronomic Institute (IAC) Plant Genetic Resources Center (Phytochemistry) Campinas SP Brazil; ^3^ Laboratory of Mass Spectrometry MS4Life São Francisco University Bragança Paulista SP Brazil; ^4^ Faculty of Pharmaceutical Science State University of Campinas Campinas SP Brazil

**Keywords:** chromatography, essential oil, hydroethanolic extract, medicinal plants, metabolomics

## Abstract

*Lippia alba* (Verbenaceae) is a native Brazilian medicinal species with varied applications, notable for the diversity of volatile chemotypes defined by the major compounds of its essential oil, with fewer studies of its nonvolatile composition. The objective of this work was to perform the chemical characterization via GC‐MS and LC‐MS of five *L. alba* individuals, collected during the rainy and dry season. The chromatographic results were analyzed via untargeted metabolomics. Four of the five individuals maintained the same major essential oil composition between the two periods evaluated and each volatile chemotype presented distinct major compounds. The nonvolatile composition, however, was similar for the five *L. alba* individuals. Some volatile and nonvolatile compounds varied between seasons, demonstrating a discrete effect of the environment on their chemical composition. These results are fundamental for the medicinal application and quality control of this species.

## Graphical abstract

*L. alba*, a Brazilian medicinal species, is known for its volatile chemotypes, with fewer studies of its nonvolatile composition. Chemical characterization via GC‐MS and LC‐MS of five individuals, during the rainy and dry season, confirm the variability of the volatile composition but the nonvolatile composition was similar, with a discrete seasonal effect. Fundamental for quality control.

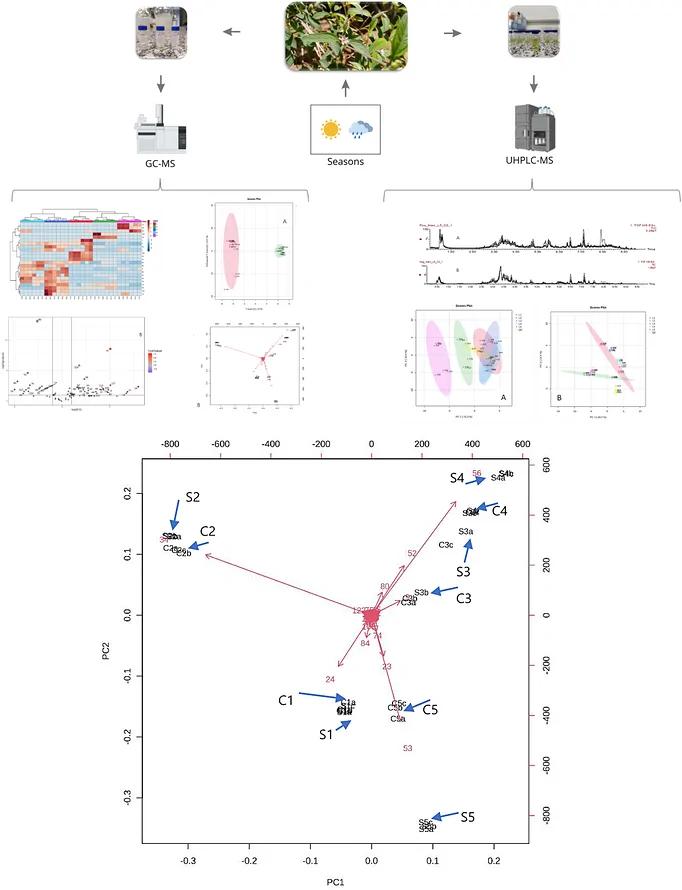

## 1 Introduction

Medicinal plants are used traditionally worldwide, with agronomic, pharmaceutical, food, and cosmetic potential [1, 2, 3, 4]. Therefore, studies ranging from taxonomic identification to chemical characterization of these plants are fundamental for their safe use [5]. As medicinal plants are living organisms, their metabolism is necessarily affected by their genes, but also by the environment [6]. Most bioactive substances in plants originate from their specialized metabolism, which is derived from metabolic pathways specific to the genus or species, but is also strongly affected by biotic and abiotic environmental factors that can influence the variety of chemical molecules produced and their quantities [6].

The Verbenaceae family has a pantropical distribution, with 36 genera and 1000 species, of which 16 genera and 300 species occur in Brazil [7]. As the *Lippia* genus contains species with known medicinal potential, studies of its species have been widely carried out [8, 9]. *L. alba* (Mill.) N.E.Br. ex Britton & P.Wilson, known as lemon balm (*cidreira*) and false melissa in Brazil, is a species of great economic interest in the biological, pharmaceutical and agronomic fields [10, 11]. This species produces important bioactive substances, such as terpenes, flavonoids, phenylpropanoids, iridoids, etc. [12, 13, 14, 15]. There are records of popular use of this species against inflammation, respiratory and gastrointestinal diseases, hypertension, depression, among others [16]. Studies in the literature point to the existence of *L. alba* chemotypes, where each chemotype is defined by the major volatile compound present in the essential oil, for example: citral, linalool, 1,8‐cineole, citral‐limonene‐carvone, myrcene and *γ*‐terpinene [1, 10, 17, 18, 19]. Medicinal applications have been suggested based on the volatile composition, rather than the nonvolatile composition.

Chromatography associated with other spectrometric techniques (hyphenated methods) are commonly used to identify these volatile chemotypes; gas chromatography coupled to mass spectrometry (GC‐MS) for the characterization of volatile compounds and liquid chromatography coupled to mass spectrometry (LC‐MS) for the nonvolatile composition [20]. These hyphenated techniques are used because they provide robust information on the composition and ensure greater accuracy in identifications [21], however, they produce a large volume of data, so specific software is necessary to extract, group and correlate data [22].

One of the promising methods for studying medicinal plants is metabolomics, which consists of the characterization and identification of chemical substances present in organisms [23]. It can be classified as untargeted (global) metabolomics, when it refers to the broad detection of compounds that may or may not be identified or targeted, which consists of the identification and quantification of compounds or classes of compounds that are already known/reported, both are important in various economic sectors [24, 25, 26]. Due to this chemical characterization, metabolomics has made it possible to find products, byproducts, and/or compounds that have beneficial activities for human health, as well as for the environment [27, 28].

Herin, we have performed the chemical characterization via GC‐MS and LC‐MS of five *L. alba* individuals (L1‐L5), which were allegedly different volatile chemotypes. The leaves were collected during the rainy and dry season to detect possible seasonal variations. The chromatographic results were analyzed via untargeted metabolomics. The purpose of this study was to determine if the nonvolatile composition of these chemotypes (originally based on volatile composition) also varied in the same manner, as well as to detect possible effects of seasons on the composition. This is the first study to analyze both the volatile and nonvolatile composition of different volatile chemotypes during two seasons in Brazil, via untargeted metabolomics. These results are fundamental for the therapeutic application and quality control of this species.

## 2 Results and Discussion

The environmental conditions were monitored for a fortnight before the collections. In December 2021 (C), the sum of precipitation in the fifteen days preceding the collection was 25.15 mm, the average temperature was 24.4°C, and the relative humidity was 63.91%. In the fifteen days preceding the collection carried out in June 2022 (S) there was no recorded rainfall, the average temperature was 19.61°C, and the relative humidity was 60.82%. Although the plants were watered on alternate days during the year, in the rainy period (C) the plants received more water as a consequence of precipitation and the relative humidity was higher, as was the temperature. Therefore, the environmental conditions should be considered as different between the two collections.

### 2.1 Volatile Composition of the Five Individuals

From the GC‐MS chromatograms of the essential oil (EO), 140 peaks were integrated, of which 66 were annotated (putatively identified). The extracted peaks (features) are numbered in the Supporting Information (Table S1). The chromatograms shown in the Supplementary Material (Figures S1–s5) indicate the most abundant compounds in the individuals.

L1 presented 1,8‐cineole (24) (C: 29.81%; S: 32.21%) as the most intense compound in the EO in both collections, followed by *α*‐guaiene (84) (C: 12.49%; S: 16.48%) and *α*‐bulnesene (108) (C: 10.01%; S: 13.13%). In both seasons the chromatograms showed a similar profile. A specimen collected the city of Catimbau—Pernambuco (PE) Brazil presented 1,8‐cineole as the major compound (70.01%), but the subsequent compounds were different from those found in our study, being *γ*‐muurolene (9.24%) and *β*‐ocimene (7.77%) [1]. This EO was tested and showed activity against termites and corn weevils (*Nasutitermes corniger* and *Sitophilus zeamais*), currently classified as pests, being proposed as a natural pesticide, but without a repellent effect.

L2 presented linalool (34) as the main component in both periods (C: 44.47%; S: 49.47%), however, the other compounds varied: being geranial (56) (4.73%) and 1,8‐cineole (24) (4.71%) in the rainy period (C) and *γ*‐muurolene (92) (7.52%) and geranial (56) (7.36%) in the dry period (S). Some authors analyzed *L. alba* EO from Santarém (PA) Brazil, this specimen contained linalool (68.31%) as the main compound in the EO followed by E‐caryophyllene (4.14%) and *γ*‐muurolene (4.13%), resembling the volatile chemotype studied herein in the dry season (S) [19].

The EO of L3 and L4 presented similar results. In individual L3, the main compounds were geranial (56) (C: 19.66%; S: 26.99%) and neral (52) (C: 18.55%; S: 20.41%), followed by myrcene (9) (C: 7.84%; S: 11.29%). L4 showed geranial (56) (C: 30.45%; S: 38.03%) and neral (52) (C: 16.78%; S: 26.86%) as major compounds in both seasons. However, in the rainy season, myrcene (9) (7.81%) was the third major compound, followed by E‐caryophyllene (80) (5.84%). In the dry season, E‐caryophyllene (80) (10.02%) showed higher proportions than myrcene (9) (8.04%). However, these four compounds stand out in L4 collected in both periods.

This volatile chemotype, denominated citral, contains two isomers: geranial and neral. A negative correlation between geranial and neral has been discussed in the literature, as these isomeric compounds share the same biosynthetic pathway, with geranyl pyrophosphate (GPP) acting as their common precursor [29]. In another study this was also observed in dry material, with geranial (32.31%), neral (23.84%) and *γ*‐murolene (7.67%) [19]. In the EO of fresh leaves, geranial (43.25%) was predominant, followed by neral (30.17%) and E‐caryophyllene (5.76%) [9], which resembles the EO present in L4 during the rainy season, showing the same sequence of major compounds. Conversely, other studies reported neral (25.88%) in a slightly higher percentage than geranial (24.09%), followed by limonene (18.53%) as major compounds [15]. Unlike L3 and L4, which presented geranial in higher proportions in both collection periods. Detected activities of the citral chemotype are antioxidant [9], vasorelaxant [30] and against *Schistosoma mansoni* [15].

L5 was the only individual in which the major compound varied between seasons, although carvone (53) stands out in the chromatogram in both. In the rainy season, carvacrol (74) (24.06%) was the main compound, followed by carvone (53) (11.92%) and limonene (23) (8.70%). In the dry season carvone (53) (52.64%) was the main compound, followed by limonene (23) (18.71%) and *γ*‐murolene (92) (11.00%). Carvacrol was not found in the EO of the other individuals, nor in the EO extracted in the dry season from individual 5. The main precursor of p‐cymene and/or limonene is geranyl diphosphate (GDP). If p‐cymene is formed, it can subsequently be converted into carvacrol. If GDP is converted into limonene, it can subsequently be transformed into carvone. This negative correlation found in the volatile composition of the two periods may be a result of environmentally modulated metabolic shifts on this biosynthetic pathway [31, 32, 33].

The carvone chemotype was observed by Barbosa et al. (2023) in EO of *L. alba* from Fortaleza (CE), Brazil, with 70% carvone and 19.2% limonene, similar to L5 in the in the dry period (S) [34]. Previous studies reported *L. alba* EO with carvone (62.86%) as the major compound, followed by limonene (26.51%) [35]. This EO, which presented toxic effects on termites (*Nasutitermes corniger*), could be a potential biological control. Other authors have characterized a *L. alba* carvone chemotype (54.57% carvone, 23.13% limonene and 4.84% alpha‐murolene) [36]. The authors suggested that the main component, carvone, could be responsible for its anxiolytic activity.

**FIGURE 1:**
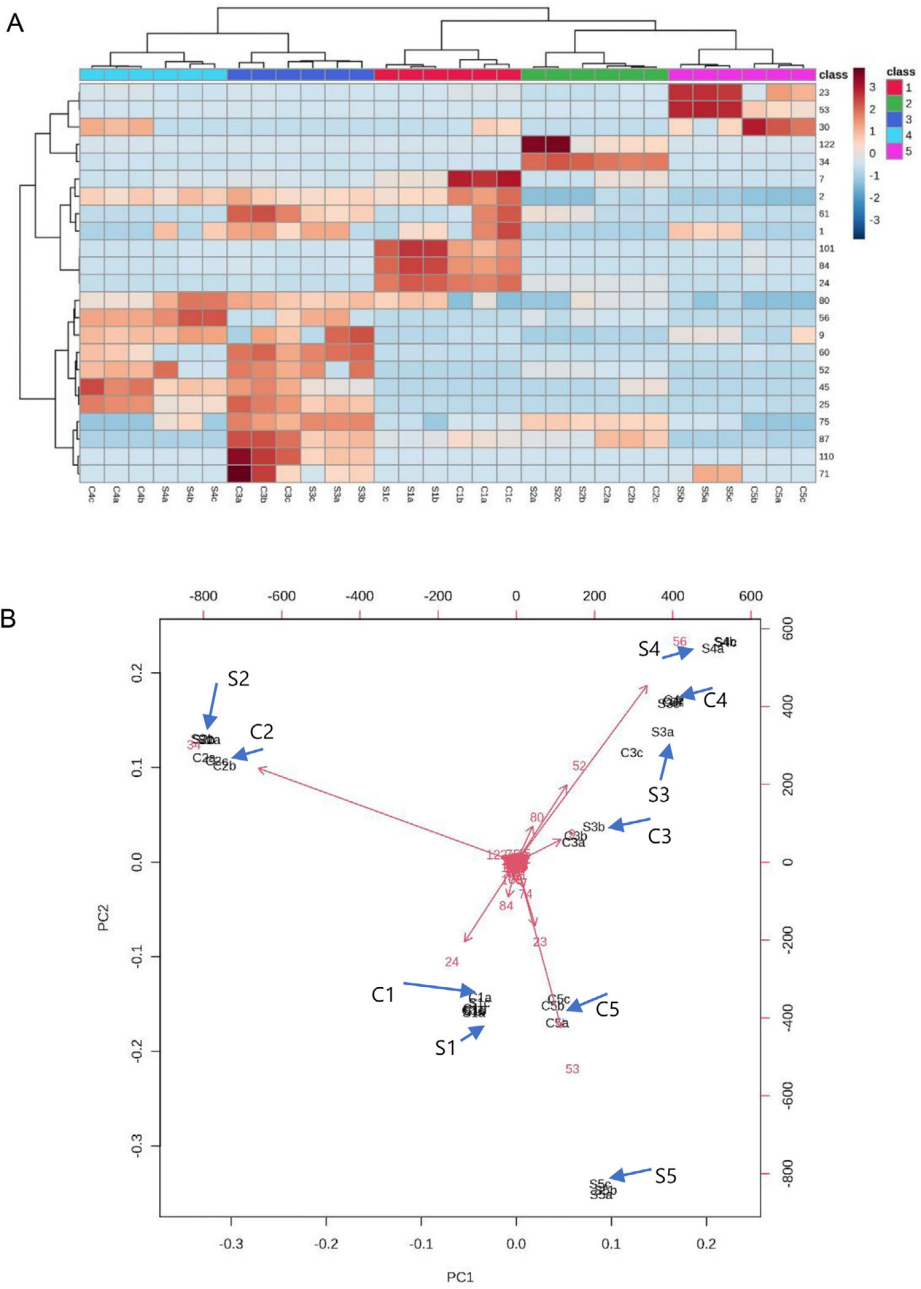
A) Heat map of the five *L. alba* individuals and their respective compounds responsible for classification. C (rainy) or S (dry): collection period; 1–5: individual number; a, b, or c: replicate of individuals. B) PCA biplot indicating the main compounds present in the essential oil of the five *L. alba* individuals responsible for the observed separations. R‐squared: 0.95037.

Most studies of *L. alba* EO involved a single collection, with some exceptions. A study evaluating several *L. alba* genotypes. The citral genotype presented the same main compounds (geranial and neral) in two seasons (summer and winter) although the other metabolites varied [18], similar to the results herein. As the main components of the EO in each volatile chemotype were apparently stable, the data were submitted to chemometric analysis, to detect minor variations. The samples were (unsurprisingly) grouped according to their volatile chemotype and main components (Figures S1–S5) and the comparison of the heatmap (Figure 1A) and PCA biplot (Figure 1B) reinforce this observation.

1,8‐cineole (24) was the main compound in both collections of L1 in and responsible for the grouping of these samples in the PCA and heatmap. Linalool (34) was the main compound both collections of L2, also responsible for its grouping and was also the most intense peak in the chromatogram. Geranial (56) and neral (52) compounds were the most intense peaks in the chromatograms of individuals 3 and 4, which were grouped in the heatmap (Figure 1A) and PCA biplot (Figure 1B). However, myrcene (9) and E‐caryophyllene (80) also influenced this grouping and 45 (trans‐4‐caranone) and 25 ((Z)‐*β*‐ocimene), which are almost imperceptible in the chromatograms, are also important in the grouping of these individuals. Carvone (53) and limonene (23) differentiate individual 5 from the others, especially in the dry season.

Some authors, however, noted the effect of growth conditions on the essential oil composition, these authors standardized the cultivation of cuttings and subsequently took the seedlings to two different fields to evaluate the difference in soil and climatic conditions of seven *L. alba* accessions [29]. They found differences between collections in the same location and in different locations; They concluded that although the main compounds did not vary, other characteristics could significantly alter the essential oil, making standardization in cultivation necessary to obtain essential oil from perennial plants, such as *L. alba*.

**FIGURE 2:**
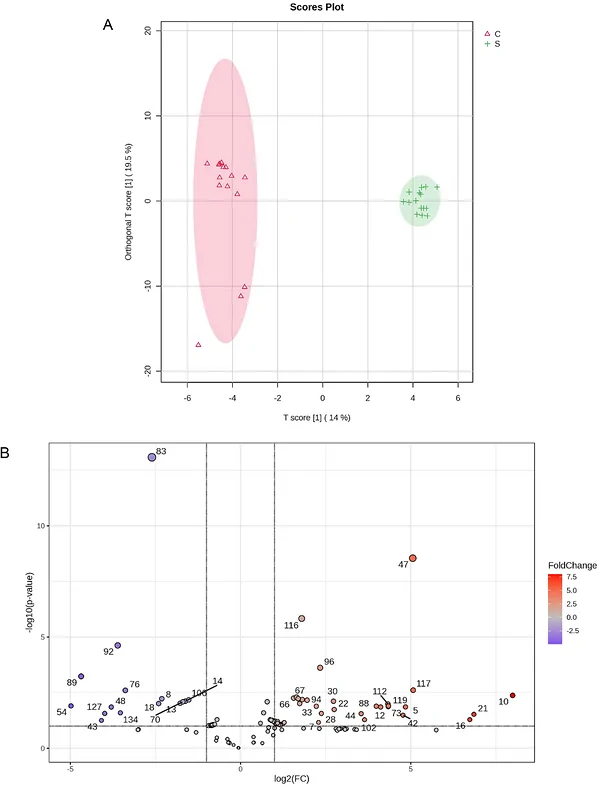
A) Orthogonal partial least squares discriminant analysis (OPLS‐DA) of the two periods of the year. C: Rainy season; S: Dry season. Q2: 0.908 *p* < 0.05 and R2Y: 0.953 *p* < 0.05. B) Volcano plot comparing the compounds that increased significantly in the dry season (blue) and in the rainy season (red).

Although the main components of the essential oil in each volatile chemotype were generally stable, the data were submitted to chemometric analysis to pinpoint variations caused by the environment, the data was divided by collection (C and S) and analyzed by OPLS‐DA (Figure 2A). The pattern differed between seasons, with the samples collected during the rainy season showing greater variation, mainly observed in individual 5 during the rainy season (C5). This result is not surprising due to the large number of peaks observed in the chromatogram of this individual, which diverged from the pattern of the other chromatograms. Individuals collected during the dry season clustered more closely. This variation in specialized (or secondary) metabolism is reported in a study, where the authors presented reports of biotic and abiotic factors that can affect the plant and its products [6]. In this study, the proportions of major compounds did not change (except for L5) when related to the collection period, which is interesting in terms of quality control of the chemical composition of this essential oil.

The volcano plot (Figure 2B) compares the profile of the samples during the rainy and dry seasons. Sixty‐five compounds were classified as significant (they increased or decreased significantly in one of the periods) and seventy‐five compounds did not fluctuate significantly between the periods. The volcano plot indicated the compounds *β*‐copaene (83), myrtenal (47) and *δ*‐cadinene (116) as the most important for differentiating the two periods.

*β*‐copaene (83) showed a decrease of approximately 2.5 times in the rainy season compared to the dry season, being found in the dry season in all individuals, with an average concentration of 0.35%. Myrtenal (47) showed an average concentration of 0.90% in the rainy season (C) and was not present in the dry season (S). Although δ‐cadinene (116) was present in both periods, it was approximately 3 times greater in the rainy season (C); showing an average concentration of 2.57% in the rainy season and 0.82% in the dry season. This result shows that, in spite of individual plant genetics, some biosynthetic pathways are common to the species and are influenced by the environment in a similar manner.

For other compounds, in addition to the variation between the two periods, variability among individuals is noted. The compound, *γ*‐murolene (92), was detected in all samples during the dry period, but only L2 and L3 during the rainy period. The compound, *α*‐amorphene (96), was detected only in L2, L3, and L4 in the rainy period whereas 9‐epi‐(E)‐caryophyllene (89) was detected only in L2, L3, and L5 in the dry period (S), and trans‐cadina‐1,4‐diene (117) was detected only in the rainy period in L1, L2, and L3.

The chromatographic profiles of the same individual were similar between the two collections, mainly in relation to the major compounds. However, when performing the chemometric analysis of the periods, it was possible to observe the presence of characteristic marker compounds. By separating the results by individual and by collection, it was possible to observe that the composition of the EO of some individuals was more affected by the collection period than others. In the PCA biplot (Figure 2B), L1 and L 2 did not show large variations between C and S. However, L3, L4, and L5 are more dispersed. These effects of the environment on the essential oil of the volatile chemotypes must be kept in mind when cuttings are planted in a different region or leaves are collected at different times of the year, even though the essential oil composition was generally stable and different for each individual plant.

### 2.2 Nonvolatile Composition of the Five Individuals

**FIGURE 3:**
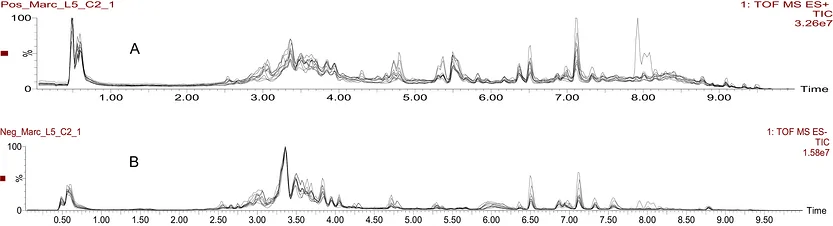
Ten overlaid UHPLC‐MS chromatograms of the five individuals (L1–L5) in two collections (C and S) in the positive (A) and negative (B) ion modes.

The chromatographic profiles of the hydroethanolic extracts of the five individuals presented a similar pattern, as noted in the overlaid chromatograms shown in Figure 3, in both the specimens (volatile chemotypes) presented mainly quantitative differences in some features. Chromatograms in both the positive and negative ion modes are shown for each for each individual (L1–L5) in Figures S6 and S7.

**FIGURE 4:**
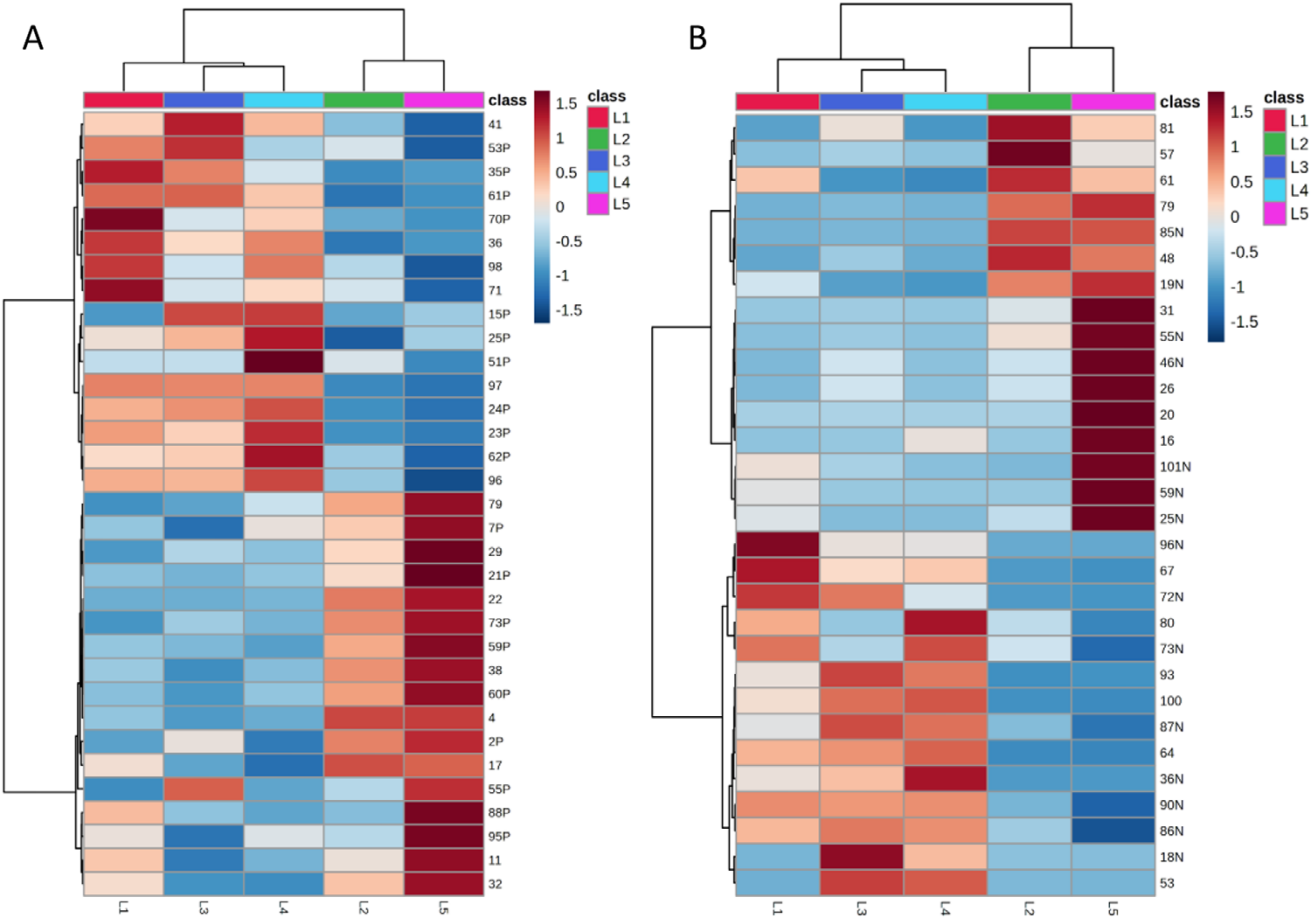
Heatmaps of (A) the 33 most important features in the positive ion mode and (B) the 30 most important features in the negative ion mode, using the PLS‐DA VIP scores. Samples L1–L5, features numbered according to Tables S2 and S3 in the Supporting Information.

The PCA scores graphs in both modes, with QC samples, are displayed in the Supporting material (Figure S8A, B) indicating the reproducibility of the analytical method. The 100 most intense features were extracted from the data in both modes and used to compare the results of the five specimens (volatile chemotypes). Initially the results were compared by grouping the results for each individual (L1–L5) as shown in Figure 4. The putative identifications are listed in the Supporting Information, Tables S2 and S3.

L3 and 4 were grouped in both modes (citral volatile chemotype), but were also similar to L1 (1,8‐cineole volatile chemotype). Putatively identified components that were more intense in these three individuals were 23P (Melilotoside—a coumarin), 24P (Plumieride—an iridoid) 25P (Verbascoside—a phenylpropanoid), 61P (Cirsilineol‐ a flavonoid) and 62P (Rosmadial ‐a diterpenoid) in the positive ion mode and 36N (Clerodendrin—a flavonoid), 86N (Hispidulin—a flavonoid), 90N (Cirsilineol—a flavonoid) and 96N (terpene glycoside) in the negative ion mode.

L2 (linalool volatile chemotype) and L5 (carvone/carvacrol volatile chemotype) were also grouped in both modes, putatively identified components that were more intense in these three individuals in the positive ion mode were 2P (FAPy‐adenine‐ oxidized DNA), 21P (Umbeliferone—a coumarin), 55P (Irigenin—flavonoid), 59P (Pectolaringenin—a flavonoid), 60P (Skullcapflavone II‐ a flavonoid), 73P (Cirsimaritin—a flavonoid) and 88P (Glyceryl linoleate—a fatty acid).In the negative ion mode 19N (Decaffeoyl verbascoside—a phenylpropanoid) and 85 N (a fatty acid) were intense in both individuals.

These results indicate that the biosynthetic pathways in all five individuals are capable of producing coumarins, flavonoids, and phenolic compounds. Verbascoside, also known as acteoside, and isoverbascoside (mass 624.2060) are phenyl propanoid isomers found in all the volatile chemotypes (25P and 32N). These compounds were identified in *L. alba* by other authors [37, 38, 39, 40], and may be considered a chemical marker of this species. Furthermore, this class of molecules possess vasorelaxant potential [41], which may contribute to the therapeutic activity of the species. Some studies of the antithrombotic effects [37] may be related to the coumarins 21P (Umbeliferone) and 23P (Melilotoside) in the HE. Furthermore, many of the flavonoids and their glycosides annotated in these hydroethanolic extracts have antioxidant potential [42].

Few authors have studied the variation in the nonvolatile composition due to the seasons. Some authors compared the hydroethanolic extracts of summer and winter collections of plants of the linalool‐1,8‐cineole and carvone‐limonene chemotypes [43]. They concluded that flavonoids were more intense in the summer (especially tricin‐7‐O‐diglucuronide) and acteoside (also known as verbascoside) was more intense in the winter collection.

**FIGURE 5:**
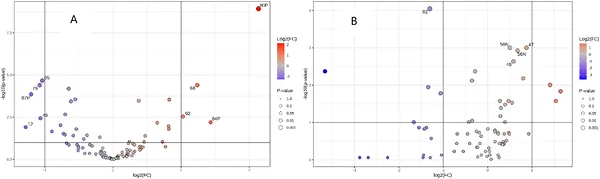
Volcano plots of (A) the features in the positive ion mode and (B) the features in the negative ion mode. Samples grouped as C and S; features numbered according to Tables S2 and S3 in the Supporting Information.

To compare the two collection periods of the five individuals, the results were grouped as C and S and compared via volcano plots. In the positive ion mode only 9 features were significantly different between groups. The identified compounds are all related to fatty acids: 80P (glyceryl 1‐linolenate) and 84P (MGMG 18:3) increased in C samples while 87 P (13‐docosenoic acid methyl ester) increased in S (Figure 5A). In the negative ion mode only eight features varied significantly, two flavonoids were identified and increased in C: 56N (Tricin diglucuronoside) and 58N (Clerodendrin) (Figure 5B). This result is in agreement with a study who observed that flavonoids increased in the summer (C was collected in December) [43]. Further studies could confirm if all the flavonoids in this species increase in the summer months, as this class is related to many of the therapeutic activities of *L. alba*.

## 3 Conclusions

Analysis of the chemical composition of five *L. alba* individuals, allegedly different volatile chemotypes, cultivated under the same conditions presented contrasting results between their volatile and nonvolatile composition. The GC‐MS analysis of the EO, four of the five individuals presented the same main compound in both collections (C and S), L1‐ 1,8‐cineole, L2‐ linalool, L3 and L4‐ citral. The main compounds in L5 result from the same biosynthetic pathway and were carvone (C) and carvacrol (S), The chromatographic profiles of the same individual were similar between the two collections, mainly in relation to the major compounds. However, when performing the chemometric analysis of the periods, it was possible to observe the presence of characteristic marker compounds. and the composition of the EO of some individuals was more affected by the collection period than others.

In contrast, the nonvolatile profile of all five individuals was overall similar, all extracts contained coumarins, flavonoids and phenolic compounds, with only quantitative differences in some features. Verbascoside, a phenylpropanoid which is characteristic of this species, was found in all the individuals. The LC‐MS analysis ofhydroethanolic extractsgrouped L3 and L4 (like the EO) together with L1, placing L1 and L5 in another group. The only significant difference between the two collection periods was an increase in flavonoids in C. In general, the nonvolatile composition varied less than the volatile composition, which these chemotypes are generally based on; and the period of collection affected the composition only marginally. These results contribute to the understanding of the chemical plasticity of *L. alba;* highlighting the importance of seasonal and genetic aspects in the standardization of plant raw materials mainly in the formulation of industrial products based on essential oils.

## 4 Experimental Section

### 4.1 Plant Material

This study was registered in the National System for the Management of Genetic Heritage and Associated Traditional Knowledge (SisGen), with the registration number: A31A878. Five individuals of *L. alba* (Mill.) N. E. Br. Ex Britton & P. ​​Wilson, obtained from the Nature's Pharmacy in the municipality of Jardinópolis—SP, were initially classified as 1, 2, 3, 4, and 5. Cuttings were rooted and planted in seventy‐kilogram pots in the field of the Institute of Biology (IB) of the State University of Campinas (UNICAMP) (22°49'11.3“S 47°04'15.4”W) with irrigation on alternate days. Their exsiccates were deposited in the Herbarium of the State University of Campinas (UEC Herbarium), obtaining the following vouchers: 1 (UEC ‐ 174416), 2 (UEC ‐ 174417), 3 (UEC ‐ 174418), 4 (UEC ‐ 174419), and 5 (UEC ‐ 174420).

The leaves used in the chemical characterization, were collected from each plant on December 17, 2021 (C) and the July 8, 2022 (S). Precipitation, temperature and relative humidity data were collected from the Center for Meteorological and Climatic Research Applied to Agriculture within the UNICAMP campus (CEPAGRI—UNICAMP). Precipitation was calculated by summing the rainfall in millimeters from the fifteen days prior to collection, while temperature and relative humidity were calculated by averaging the rainfall from the fifteen days prior to collection.

### 4.2 Essential Oil Extraction (volatile composition)

To obtain the essential oil, an extraction was performed per individual from 60 g of leaves (at different stages of development) of L. alba, 1.2 L of distilled water was added and ground for 7 (seven) seconds using a blender. Subsequently, this mixture was placed in a flask, attached to the Clevenger apparatus and subjected to the hydrodistillation process for 3 h.

### 4.3 GC‐MS Analysis and Data Extraction

GC‐MS analysis was performed on an Agilent Model 7890A gas chromatograph (Agilent Technologies, CA, USA) with an HP‐5 MS Agilent J&W column (20 m × 0.25 mm × 0.25 µm) coupled to a mass spectrometer with electron ionization (70 eV). Analytical conditions: injector temperature of 220°C, interface temperature of 240°C. The oven temperature started at 60°C, increasing 3°C/min, up to 246°C. Helium was used as the carrier gas at a flow rate of 1 mL/min [44].

The essential oils were diluted in a 1:10 ratio in ethyl acetate, and 1 µL of sample was injected with a 1:20 split. The oils were injected in triplicate. A series of alkanes (C8–C20) were also injected to aid in the identification of compounds through the calculation of the linear retention index using the equation:

IRL=100×(n+(RT−RTn)/(RTN−RTn))

Where, IRL: linear retention index; RT: retention time of the compound of interest; RTn: retention time of the preceding hydrocarbon; RTN: retention time of the subsequent hydrocarbon; n: number of carbons in the preceding hydrocarbon.

Features (*m/z* and retention time) were extracted from the chromatograms using the MS‐DIAL (MS Data Independent Analysis Liaison) software, followed by manual data curation. Compound identification was further performed by comparing the fragmentation spectrum with the NIST (National Institute of Standards and Technology) library and the MassBank of North America database, as well as by calculating the retention index and comparing the values ​​with the literature [44]. Data was organized in Excel tables of samples vs features, registering feature intensity for each sample. No QC samples were prepared for the essential oil analysis for technical reasons.

### 4.4 Hydroethanolic Extracts (Nonvolatile Composition)

Collected leaf samples were freeze dried, 2 mg of each sample was weighed and extracted in 1 mL 50% ethanol / water solution in an ultrasonic bath for 30 min, then filtered. QC samples were prepared pooling 50 µL of each vial.

### 4.5 LC‐MS Analysis and Data Extraction

Ultra‐high‐performance liquid chromatography coupled to mass spectrometry (UHPLC‐MS) was performed on an Acquity UPLC chromatograph coupled to a TOF Mass spectrometer (Waters Manchester, England), using a C18 HSS T3 column (2.1 mm x 50.0 mm x 1.8 µm). The mobile phase consisted of ultrapure water with 0.1% formic acid (A) and acetonitrile (B) with a gradient: starting with 5% B, reaching 100% B in 8 min, maintaining it for up to 9.0 min, returning to the initial conditions in 9.1 min and remaining for up to 10 min to stabilize the column. The volume of the injected sample was 5 µL. The MS spectrometer operated in positive and negative mode with electrospray ionization under the following conditions: capillary +3.00 kV, cone +30 V, source temperature of 150°C and desolvation temperature of 300°C in full scan mode *m/z* 100–1000. QC samples were injected once every 10 samples. The chromatograms were compared, aligned and the features in both positive and negative ion modes extracted using Progenesis software. Data was organized in Excel tables of samples vs features, registering feature intensity for each sample.

### 4.6 Statistical Analysis

GC‐MS and LC‐MS features were treated using online MetaboAnalyst software. For the analysis by collection, two classes (C and S) were considered, where all samples (five per period) and their triplicates were classified as group C for the rainy period and group S for the dry period. The data was normalized by sum and mean‐centered. PCA, PLS‐DA and OPLS‐DA were performed, as well as volcano plot analysis to compare the two collections and detect significantly different features. For analysis by individual, five classes were considered (L1, L2, L3, L4 and L5), wherein the results for each individual in both seasons were grouped. The data was normalized by sum and autoscaled PCA, PLS‐DA and heatmap analyses were performed to detect significantly different features (P<0.05%) between individuals.

To provide a clear overview of the data processing strategy, the untargeted metabolomics workflow was structured into sequential operational stages, as follows: GC‐MS/UHPLC‐MS Feature extraction → Data processing (MetaboAnalyst) → Normalization by sum → Data scaling by Mean‐centering → Statistical Analysis (PCA, PLS‐DA, OPLS‐DA, Volcano plot and Heatmap) → Metabolite annotation (MS‐DIAL, NIST, MassBank of North America database & Adams libraries).

## Author Contributions

**Marcelino Santiago Barroso Neto**: conceptualization, data curation, formal analysis, methodology, Writing – original draft and reviewing. **Erilene Nayrã Mendonça Brito Lopes**: investigation, methodology. **Marcia Ortiz Mayo Marques**: resouces, supervision. **Alex Aparecido Rosini Silva**: methodology, softwate, validation. **Patricia de Oliveira Carvalho**: resources, supervision. **Alexandra Christine Helena Frankland Sawaya**: writing – original draft and reviewing, supervision, conceptualization.

## Conflicts of Interest

The authors declare no conflicts of interest.

## Supporting information




**Supporting File 1**: cbdv71664‐sup‐0001‐SuppMat.docx

## Data Availability

Data will be made available via the corresponding author upon reasonable request.
