# Supplementary material for: Seasonal Effect on the Chemical Composition of Lippia alba Volatile Chemotypes
Source: Chem Biodivers. 2026 Aug 31;23(9):e71664. doi: 10.1002/cbdv.71664 (PMC13529227; doi:10.1002/cbdv.71664)
Supplement: Supplementary file 1 [file CBDV-23-e71664-s001.docx]

Supplementary material

**Figure S1.** GC-MS chromatograms of EO Plant 1 in the rainy (C) and dry (S) season and the most intense components.

 **Figure *S*2.** GC-MS of EO Plant 2 in the rainy (C) and dry (S) season an*d* the most intense components.

**Figure S3.** GC-MS of EO Plant 3 in the rainy (C) and dry (S) season and the most intense components.

**Figure S4.** GC-MS of EO Plant 4 in the rainy (C) and dry (S) season and the most intense components.

**Figure S5.** GC-MS of EO Plant 5 in the rainy (C) and dry (S) season and the most intense components.

**Figure S6.** LC-MS chromatograms of the extracts in the positive ion mode. A) L5S, B) L5C, C) L4S, D) L4C, E) L3S, F) L3C, G) L2S, H) L2C, I) L1S and J) L1C.

**Figure S7.** LC-MS chromatograms of the extracts in the negative ion mode. A) L5S, B) L5C, C) L4S, D) L4C, E) L3S, F) L3C, G) L2S, H) L2C, I) L1S and J) L1C.

**Figure S8.** PCA scores graph of the UHPLC-MS results in the (A) positive and (B) negative ion modes (with QC samples).

**Table S1.** Percentage of total intensity of peaks extracted from the GC-MS chromatograms of the *L. alba* essential. Peak No.: peak number; RT: retention time; RI: retention index; RIA: Adams retention index (2017); C: collection during the rainy season; S: collection during the dry season; 1-5: individual plant number.

| **Peak No.** | **RT** | **Putative identification** | **RI** | **RIA** | **C1** | **S1** | **C2** | **S2** | **C3** | **S3** | **C4** | **S4** | **C5** | **S5** |
| --- | --- | --- | --- | --- | --- | --- | --- | --- | --- | --- | --- | --- | --- | --- |
| 1 | 5.24 | α-thujene | 925 | 924 | 0.72 | 0.29 | - | - | 0.40 | 0.48 | - | 0.36 | - | 0.39 |
| 2 | 5.42 | α-pinene | 932 | 932 | 1.64 | 0.96 | 0.43 | - | 1.05 | 0.92 | 1.10 | 1.00 | - | 0.26 |
| 5 | 5.98 | Thuja-2,4(10)-diene | 954 | 953 | - | - | - | - | 0.46 | - | 0.44 | - | - | - |
| 7 | 6.46 | Sabinene | 973 | 969 | 6.08 | 1.07 | 1.06 | - | 0.38 | 0.19 | - | 0.27 | - | - |
| 8 | 6.56 | β-pinene | 977 | 974 | 1.34 | 4.41 | - | 0.63 | 0.31 | 0.4 | - | 0.70 | 0.25 | 2.04 |
| 9 | 6.91 | Myrcene | 990 | 988 | 1.61 | 0.80 | 0.65 | - | 7.84 | 11.29 | 7.81 | 8.04 | 6.09 | 4.12 |
| 11 | 7.07 | Mentha-1(7),8-diene <meta-> | 997 | 1000 | - | - | - | - | - | - | - | - | 0.42 | - |
| 16 | 7.36 | α-felandrene | 1006 | 1002 | - | - | - | - | - | - | 0.5 | - | 0.89 | - |
| 19 | 7.55 | (E)-hex-2-enyl acetate | 1012 | 1010 | - | - | - | - | - | - | - | - | 0.73 | - |
| 21 | 7.73 | α-terpinene | 1017 | 1014 | 0.87 | - | - | - | - | - | 0.69 | - | 5.59 | - |
| 22 | 8.05 | o-Cymene | 1026 | 1022 | - | - | - | - | 0.43 | 0.26 | 4.09 | 0.28 | 1.45 | - |
| 23 | 8.12 | Limonene | 1028 | 1024 | 1.80 | 0.76 | 0.85 | - | 0.90 | 0.57 | 1.56 | 0.55 | 8.70 | 18.71 |
| 24 | 8.24 | 1,8-Cineole | 1033 | 1026 | 29.81 | 32.21 | 4.71 | 5.21 | 0.75 | - | - | - | 0.50 | - |
| 25 | 8.39 | (Z)-β-ocimene | 1037 | 1032 | - | - | - | - | 0.72 | 0.46 | 0.78 | 0.31 | - | - |
| 27 | 8.75 | (E)-β-ocimene | 1047 | 1044 | 1.20 | 0.81 | 1.09 | 0.77 | 1.19 | 0.89 | 1.73 | 0.97 | 0.35 | - |
| 28 | 9.14 | γ-terpinene | 1059 | 1054 | 1.08 | 0.22 | - | - | 0.41 | 0.30 | 0.81 | - | 2.79 | 0.33 |
| 30 | 9.91 | Terpinolene | 1090 | 1086 | 0.68 | - | - | - | - | - | 1.14 | - | 1.58 | 0.77 |
| 32 | 10.14 | ρ-mentha-2,4(8)-diene | 1088 | 1085 | - | - | - | - | - | - | - | - | 0.75 | - |
| 33 | 10.45 | 2,5-Dimethylstyrene | 1097 | 1099 | - | - | - | - | - | - | 1.02 | - | 0.37 | - |
| 34 | 11.06 | Linalool | 1115 | 1095 | 0.67 | 0.27 | 44.47 | 49.47 | 1.04 | 0.37 | 0.74 | - | - | 0.69 |
| 35 | 11.19 | ρ-Mentha-1,3,8-triene | 1116 | 1108 | - | - | - | - | - | - | 0.96 | - | - | - |
| 45 | 14.14 | trans-4-caranone | 1191 | 1196 | - | - | 0.67 | - | 1.45 | 0.54 | 2.11 | 0.99 | - | - |
| 47 | 14.83 | Myrtenal | 1208 | 1195 | 0.91 | - | 0.82 | - | 1.3 | - | 1.06 | - | 0.44 | - |
| 52 | 16.69 | Neral | 1253 | 1235 | - | - | 4.00 | 5.64 | 18.55 | 20.41 | 16.78 | 26.86 | - | - |
| 53 | 16.95 | Carvone | 1259 | 1239 | - | - | - | - | - | - | - | - | 11.92 | 52.64 |
| 56 | 17.92 | Geranial | 1284 | 1264 | - | - | 4.73 | 7.36 | 19.66 | 26.99 | 30.45 | 38.03 | - | - |
| 58 | 18.76 | Carvacrol ethyl ether | 1303 | 1297 | - | - | - | - | - | - | - | - | 2.11 | - |
| 60 | 19.95 | Myrtenyl acetate | 1332 | 1324 | - | - | - | - | 1.34 | 1.45 | 0.87 | - | - | - |
| 61 | 20.61 | α-cubebene | 1348 | 1348 | 1.03 | - | - | 0.32 | 1.05 | 0.48 | - | - | - | - |
| 67 | 21.70 | α-ylangene | 1375 | 1373 | - | - | 1.11 | - | 0.99 | - | - | - | - | - |
| 70 | 21.90 | α-copaene | 1380 | 1374 | - | - | - | 0.48 | - | 0.32 | - | - | - | - |
| 71 | 22.08 | β-bourbonene | 1384 | 1387 | - | - | - | - | 1.04 | 0.38 | - | - | - | 0.74 |
| 73 | 22.33 | Geranyl acetate | 1390 | 1379 | - | - | - | - | 1.33 | - | 1.74 | - | - | - |
| 74 | 22.39 | Carvacrol | 1392 | 1298 | - | - | - | - | - | - | - | - | 24.06 | - |
| 75 | 22.41 | β-elemene | 1392 | 1389 | 0.93 | 0.67 | 2.00 | 2.06 | 2.57 | 2.87 | - | 1.57 | - | 0.94 |
| 76 | 22.50 | β-cubebene | 1394 | 1387 | - | 0.84 | - | 0.67 | - | 2.74 | - | 0.47 | - | - |
| 77 | 23.13 | Sesquithujene | 1410 | 1405 | - | 0.34 | - | - | - | - | - | - | - | - |
| 79 | 23.40 | α-cedrene | 1417 | 1410 | - | 0.46 | - | - | - | - | - | - | - | - |
| 80 | 23.51 | E-caryophyllene | 1420 | 1417 | 5.05 | 7.38 | 4.39 | 5.44 | 7.81 | 7.00 | 5.84 | 10.02 | - | 2.39 |
| 83 | 24.08 | β-copaene | 1434 | 1430 | - | 0.34 | - | 0.30 | - | 0.42 | - | 0.38 | - | 0.33 |
| 84 | 24.27 | α-guaiene | 1439 | 1437 | 12.49 | 16.48 | - | - | - | - | - | - | - | - |
| 85 | 24.28 | γ-elemene | 1439 | 1434 | - | - | - | 0.56 | - | - | - | - | - | - |
| 86 | 24.88 | α-humulene | 1454 | 1452 | 1.03 | 0.74 | 1.11 | 0.65 | 0.82 | 0.42 | 0.88 | 0.56 | - | 0.20 |
| 87 | 25,00 | (E)-β-farnesene | 1457 | 1454 | 0.59 | 0.4 | 0.99 | 0.43 | 1.46 | 0.85 | - | - | - | - |
| 89 | 25.35 | <9-epi-(E)-caryophyllene | 1466 | 1464 | - | - | - | 0.36 | - | 0.45 | - | - | - | 0.39 |
| 90 | 25.67 | Cadina-1(6),4-diene <trans-> | 1474 | 1475 | 1.00 | - | - | 0.36 | 0.66 | 0.29 | - | - | - | 0.24 |
| 91 | 25.75 | β-chamigrene | 1476 | 1476 | - | - | - | - | - | - | 0.95 | - | - | - |
| 92 | 25.81 | γ-muurolene | 1477 | 1478 | - | 0.57 | 1.17 | 7.52 | 1.34 | 7.11 | - | 2.66 | - | 11.00 |
| 94 | 25.99 | Germacrene D | 1482 | 1480 | - | - | 1.29 | - | 1.64 | - | - | - | - | - |
| 95 | 26.12 | ar-curcumene | 1485 | 1479 | 0.80 | - | - | - | - | - | - | - | - | - |
| 96 | 26.20 | α-amorphene | 1487 | 1483 | - | - | 1.02 | - | 1.63 | - | 1.27 | - | - | - |
| 98 | 26.37 | δ-selinene | 1491 | 1492 | - | - | - | - | - | - | - | - | 1.69 | - |
| 101 | 26.55 | α-zingiberene | 1496 | 1493 | 2.17 | 3.37 | - | - | - | - | - | - | - | - |
| 103 | 26.68 | Epizonarene | 1499 | 1501 | - | - | - | - | - | - | - | - | 1.59 | - |
| 105 | 26.75 | α-muurolene | 1501 | 1500 | 0.93 | 0.84 | 0.77 | 0.38 | 0.96 | 0.40 | 0.63 | 0.37 | 0.74 | 0.29 |
| 106 | 26.81 | Bicyclogermacrene | 1503 | 1500 | - | - | - | - | - | 0.32 | - | - | - | 0.38 |
| 108 | 26.98 | α-bulnesene | 1507 | 1509 | 10.01 | 13.13 | - | 0.71 | - | 1.08 | - | 0.65 | - | 0.41 |
| 110 | 27.05 | <(E,E)-α-farnesene | 1509 | 1505 | - | - | - | - | 1.29 | 0.52 | - | - | - | - |
| 112 | 27.30 | γ-cadinene | 1516 | 1513 | - | - | 1.51 | - | 2.13 | - | - | - | - | 0.27 |
| 115 | 27.52 | Nootkatone | 1522 | 1517 | - | 0.33 | - | - | - | - | - | - | - | - |
| 116 | 27.66 | δ-cadinene | 1525 | 1522 | 2.42 | 1.5 | 2.64 | 0.98 | 3.25 | 0.75 | 1.53 | 0.28 | 3.03 | 0.60 |
| 117 | 28,00 | Cadina-1,4-diene <trans-> | 1534 | 1533 | 1.7 | - | 0.57 | - | 0.88 | - | - | - | - | - |
| 119 | 28.21 | α-cadinene | 1539 | 1537 | - | - | 0.97 | - | 0.83 | - | - | - | - | - |
| 120 | 28.34 | Selina-3,7(11)-diene | 1543 | 1545 | - | - | 1.92 | - | - | - | - | - | - | - |
| 122 | 28.96 | Germacrene B | 1559 | 1559 | - | - | 1.03 | 5.08 | - | - | - | - | - | - |
| 136 | 36.56 | Muurolene <14-hydroxi-α-> | 1772 | 1779 | - | - | - | 0.52 | - | - | - | - | - | - |

Table S2. Putatively identified features of peaks extracted from the LC-MS positive ion mode chromatograms of the *L. alba* HE with number; feature, chemical class, chemical formula, name and reference (data bank or literature reference).

| **Number** | **Formula** | **Feature positive** | **Class** | **Putative id** | **Ref.** |
| --- | --- | --- | --- | --- | --- |
| 2P | C5H7N5O | 0,56_136,0625m/z | oxidized DNA | FAPy-adenine | data bank |
| 3P | C5H9NO4 | 0,56_147,0536n | amino acid | Glutamic acid | data bank |
| 5P | C12H22O11 | 0,56_381,0795m/z | sugar | Disaccharide | data bank |
| 6P | C24H42O21 | 0,56_666,2226n | sugar | Tetrasaccharides (hex-hex-hex-hex) | data bank |
| 7P | C7H15NO3 | 0,59_161,1059n | metabolite | L-carnitine | data bank |
| 8P | C11H12O4 | 2,53_208,0744n | phenylpropanoid | Sinapyl aldehyde | data bank |
| 9P | C27H26O18 | 2,85_638,2083m/z | flavonoid | Flavone base + 4O, O-hexa-hexa | data bank |
| 10P | C27H26O18 | 2,99_639,1195m/z | flavonoid | Luteolin-7-O-diglucuronide | [45] |
| 13P | C15H10O6 | 3,06_287,0558m/z | flavonoid | 4',5,7-trihydroxyflavonol | data bank |
| 14P | C27H30O18 | 3,06_625,1403m/z | flavonoid | Flavonol base + 5O, O-Hex, O-Hex | data bank |
| 15P | C27H26O17 | 3,22_623,1249m/z | flavonoid | Clerodendrin | [38] [43] |
| 18P | C28H28O18 | 3,26_653,1368m/z | flavonoid | Chrysoeriol-7-O-diglucuronide | [38] [43] |
| 19P | C29H30O19 | 3,26_683,1456m/z | flavonoid | Tricin-7-O-diglucuronoside | [38] [43] |
| 21P | C9H6O3 | 3,36_162,0324n | coumarin | Umbelliferone or isomers | data bank |
| 23P | C15H18O8 | 3,36_309,0976m/z | coumarin | Melilotoside; | data bank |
| 24P | C21H26O12 | 3,36_471,1499m/z | iridoid | Plumieride | data bank |
| 25P | C29H36O15 | 3,36_624,2060n | phenylpropanoid | Isoverbascoside/ isoacteoside | [37] [38] [39] [40][45] |
| 26P | C15H10O6 | 3,39_286,0481n | flavonoid | Luteolin | data bank |
| 27P | C21H20O11 | 3,39_449,1080m/z | flavonoid | Luteolin-7-glucoside | data bank |
| 31P | C29H30O19 | 3,45_665,1373m/z | flavonoid | Tricin diglucuronoside | data bank |
| 35P | C30H32O20 | 3,50_695,1473m/z | flavonoid | Quercetin -malonyl-glucoside- 7-O-glucoside | data bank |
| 37P | C10H12O5 | 3,52_213,0764m/z | phenolic | Asaronic acid | data bank |
| 40P | C15H10O5 | 3,64_271,0607m/z | flavonoid | Apigenin | [37] [40] |
| 42P | C31H38O16 | 3,64_666,2297n | phenylethanoid | 2-Acetyl acteoside | [38] |
| 45P | C23H22O13 | 3,69_507,1137m/z | flavonoid | Tricin-7-O-glucuronide or isomers | [38] [43] |
| 50P | C10H12O4 | 3,92_196,0743n | phenolic | Dihydroferulic acid | data bank |
| 51P | C10H8O3 | 3,94_177,0553m/z | coumarin | 4-Methylumbelliferone | data bank |
| 52P | C16H18O8 | 3,94_338,1008n | coumarin | Gerberinside | data bank |
| 53P | C30H46O3 | 4,70_454,3443n | triterpenoid | Triterpenic acid | data bank |
| 54P | C16H12O6 | 4,72_301,0711m/z | flavonoid | 6-Methoxyapigenin= Hispidulin | [37] [40] |
| 55P | C18H16O8 | 4,74_361,0920m/z | flavonoid | Irigenin, Jaceidin or isomers | data bank |
| 57P | C17H14O7 | 4,79_331,0815m/z | flavonoid | 5,7,4`-Trihydroxy-3,6-dimethoxyflavone | [37] [40] |
| 59P | C17H14O6 | 5,30_314,0793n | flavonoid | Pectolinarigenin | [37] [40] |
| 60P | C19H20O9 | 5,32_375,1076m/z | flavonoid | Skullcapflavone II or Isomers | data bank |
| 61P | C18H16O7 | 5,37_344,0898n | flavonoid | Cirsilineol | data bank |
| 62P | C20H24O5 | 5,37_345,1694m/z | diterpene | Rosmadial | data bank |
| 63P | C18H28O4 | 5,48_308,1991n | phenolic | 2-(3-(8-Hydroxyoctyl) phenoxy) -2-methylpropanoic acid | data bank |
| 66P | C18H39NO3 | 5,53_318,3005m/z | sphingoid FA | Phytosphingosine | data bank |
| 69P | C19H18O8 | 5,60_375,1076m/z | flavonoid | Casticin; Vitexicarpin; | data bank |
| 70P | C21H34O4 | 5,65_350,2458n | phenolic | Gingerol isomer | data bank |
| 73P | C17H14O6 | 5,83_315,0866m/z | flavonoid | Cirsimaritin | data bank |
| 74P | C20H28O2 | 6,13_301,2159m/z | diterpenoid | Kaurenoic or Grandiflorenic Acid Isomer | data bank |
| 75P | C18H39NO2 | 6,16_302,3058m/z | sphingoid FA | Dihydrosphingosine; | data bank |
| 76P | C24H30O6 | 6,37_414,2044n | xanteno | Myrtucommulone B | data bank |
| 78P | C21H34O4 | 6,42_351,2531m/z | phenolic | Gingerol isomer | data bank |
| 80P | C21H36O4 | 6,50_352,2615n | glicerolipid | Glyceryl-1-Linoleate isomer | data bank |
| 81P | C33H56O14 | 6,50_676,3682n | glicerolipid | DGMG 18:3 | data bank |
| 83P | C18H28O2 | 6,88_276,2093n | fatty acid | Stearidonic acid (18:4(N-3)) | data bank |
| 84P | C27H46O9 | 7,11_514,3145n | glicerolipid | MGMG 18:3; | data bank |
| 86P | C18H30O2 | 7,13_261,2218m/z | fatty acid | Pinolenic Acid; | data bank |
| 87P | C23H44O2 | 7,13_352,3348n | fatty acid | 13-Docosenoic acid methyl esther | data bank |
| 88P | C21H36O4 | 7,13_353,2688m/z | fatty acid | Glyceryl-1-Linoleate isomer | data bank |
| 94P | C30H46O5 | 8,08_469,3312m/z | triterpene | Ceanothic acid | data bank |

Table S3 Putatively identified features of peaks extracted from the LC-MS negative ion mode chromatograms of the *L. alba* HE with number; chemical formula, feature, chemical class, name and reference (data bank or literature).

| **Number** | **Formula** | **FEATURE** | **Class** | **Putative id** | **Ref.** |
| --- | --- | --- | --- | --- | --- |
| 4N | C12H22O11 | 0,54_341,1086m/z | sugar | Dissacharide | data bank |
| 6N | C5H10O6 | 0,56_165,0400m/z | sugar | Ribonic acid/ arabinonic acid | data bank |
| 8N | C6H12O7 | 0,56_196,0579n | sugar | Galactonic or glucionic acid | data bank |
| 9N | C12H22O11 | 0,56_377,0853m/z | sugar | Dissacharide | data bank |
| 10N | C6H12O6 | 0,58_179,0556m/z | sugar | Hexose | data bank |
| 12N | C6H8O7 | 0,59_192,0264n | organic acid | Isocitric acid; Citric acid | data bank |
| 17N | C16H24O11 | 1,49_391,1239m/z | iridoid | Shanzhiside | [37] [40] |
| 18N | C16H22O10 | 1,61_373,1135m/z | iridoid | Swertiamarin or tgeniposidic acid | [12] [37] [38] [40] |
| 19N | C20H30O12 | 2,21_461,1657m/z | phenylpropanoid | Decaffeoyl-verbascoside | [37] [38], [40] |
| 23N | C16H22O11 | 2,67_390,1156n | iridoid | Scandoside; oleoside; theveside | [38], [39] |
| 25N | C18H28O9 | 2,90_388,1727n | organic acid glucoside | Tuberonic acid glucoside | data bank |
| 29N | C27H26O18 | 3,01_637,1056m/z | flavonoid | Luteolin-7-O-diglucuronide | [45] |
| 30N | C29H36O16 | 3,01_639,1936m/z | phenyletanoid | Β-hydroxyacteoside | [37] [38] [40] |
| 32N | C27H30O18 | 3,06_623,1264m/z | phenylpropanoid | Verbascoside | [37] [38] [39] [40] |
| 36N | C27H26O17 | 3,22_621,1107m/z | flavonoid | Clerodendrin | [38] [39] [43] |
| 40N | C31H38O16 | 3,26_665,2227m/z | phenylethanoid | 2-Acetylverbascoside | [38] |
| 41N | C29H30O19 | 3,26_681,1318m/z | flavonoid | Tricin-7-O-diglucuronoside | [38] [43], |
| 42N | C28H28O18 | 3,27_651,1216m/z | flavonoid | Chrysoeriol-7-O-diglucuronide | [38] [39] [43] |
| 43N | C28H34O15 | 3,29_609,1829m/z | phenylethanoid | Calceolarioside E | [37] [38] [39] [40] |
| 45N | C35H46O19 | 3,29_769,2578m/z | phenylpropanoid | Poliumoside; Forsythoside G | data bank |
| 46N | C28H28O18 | 3,32_651,1990m/z | flavonoid | Chrysoeriol-7-4-diglucuronide | data bank |
| 49N | C29H36O15 | 3,36_623,2989m/z | phenyl propanoid | Isoverbascoside | [37] [38] [39] [40] |
| 54N | C21H20O11 | 3,39_448,0999n | flavonoid | Luteolin-7-glucoside | data bank |
| 55N | C23H28O9 | 3,39_448,1749n | lignan | Aequilabrine B | [46] |
| 56N | C29H30O19 | 3,44_663,1212m/z | flavonoid | Tricin diglucuronoside | data bank |
| 58N | C27H26O17 | 3,49_621,1831m/z | flavonoid | Clerodendrin isomer | [38] [39] [43] |
| 59N | C38H36O11 | 3,49_667,2191m/z | lignan | Threo ou erythro-Dadahol B | data bank |
| 62N | C15H22O10 | 3,49_723,2332m/z | iridoid | Catalpol | data bank |
| 63N | C30H32O20 | 3,50_693,1323m/z | flavonoid | Quercetin malonyl-glucoside- 7-O-glucoside | data bank |
| 68N | C28H34O15 | 3,54_609,1837m/z | phenylpropanoid | Isonuomioside | [37] [38] [39] [40] |
| 69N | C29H36O14 | 3,55_607,2029m/z | phenylpropanoid | Lipedoside A | data bank |
| 71N | C15H18O8, | 3,59_651,1925m/z | coumarin | Trans-Melilotoside | data bank |
| 72N | C30H38O17 | 3,61_637,2130m/z | phenylethanoid | Leucosceptoside A | [38] |
| 73N | C21H20O10 | 3,64_431,0974m/z | flavonoid | Vitexin; Isovitexin isomers | data bank |
| 74N | C31H38O16 | 3,64_665,2091m/z | phenylethanoid | 2 Acetyl acteoside | [39] |
| 76N | C23H22O13 | 3,69_505,0986m/z | flavonoid | Tricin-7-O-glucuronide isomers | [38] [43] |
| 78N | C32H40O17 | 3,78_695,2207m/z | iridoid | Ncgc00386112-01 | data bank |
| 83N | C31H40O15 | 3,96_651,2295m/z | phenylpropanoid | Brachynoside or Martynoside | [37] [38] [40] |
| 85N | C18H32O5 | 4,70_327,2170m/z | fatty acid | (10E,15Z)-9,12,13-trihydroxyoctadeca-10,15-dienoic acid | data bank |
| 86N | C16H12O6 | 4,72_299,0556m/z | flavonoid | Hispidulin isomers | [37] [40] |
| 87N | C17H14O7 | 4,80_329,0660m/z | flavonoid | 5,7,4`-trihydroxy-3,6-dimethoxyflavone | [37] [40] |
| 89N | C17H14O6 | 5,30_313,0711m/z | flavonoid | Pectolinarigenin | [37] [40] |
| 90N | C18H16O7 | 5,37_343,0816m/z | flavonoid | Cirsilineol | data bank |
| 95N | C18H32O4 | 6,86_293,2117m/z | fatty acid | (9Z,12E)-15,16-dihydroxyoctadeca-9,12-dienoic acid | data bank |
| 96N | C27H46O9 | 7,13_514,3136n | fatty acid | 9,12,15-Octadecatrienoic acid, | data bank |
| 99N | C16H30O8 | 7,32_699,3822m/z | terpene glycoside | Hexose + C10H19O2 | data bank |
| 101N | C30H46O4 | 8,01_469,3320m/z | triterpenic acid | Triterpenic acids | data bank |
